# Computation of the Characteristic Parameters of Coaxial Waveguides Used in Precision Sensors

**DOI:** 10.3390/s23042324

**Published:** 2023-02-20

**Authors:** Krzysztof Kubiczek

**Affiliations:** Department of Measurement Science, Electronics and Control, Silesian University of Technology, 44-100 Gliwice, Poland; krzysztof.kubiczek@polsl.pl

**Keywords:** characteristic impedance, numerical stability, modified scaled Bessel functions, thermal voltage converters, coaxial waveguides

## Abstract

This paper presents a fast and computationally stable analytical algorithm used to perform the characteristic impedance of cylindrical multilayer waveguides used in high-precision sensors and apparatuses. Most of the algorithms used for the calculation of the characteristic impedance of those waveguides are based on approximations. Their application is limited to waveguides with a certain (usually small) number of layers. There may be insufficient layers, especially when coaxial waveguides are part of a precise measurement device. This article presents a numerically stable analytical algorithm using modified scaled Bessel functions to perform the characteristic impedance and the components of cylindrical coaxial multilayer waveguides. The results achieved by the extracted algorithm were compared to the results obtained by simulation using finite element method (FEM) software and the current method, whose main drawback is the fictive sublayers, which significantly increase the computation time. The excellent agreement between the results confirmed the precision of the algorithm and the time required for the calculation was reduced several times.

## 1. Introduction

Cylindrical coaxial waveguides and cables are currently used in many different applications, from antenna feeders to high-precision wideband measuring instruments, sensors and standards [1,2,3,4,5]. These include calculable thermal converters used in voltage AC-DC transfer [6,7], which usually require a multilayer coaxial waveguide to feed the microwave heater fixed to the multijunction thermocouple. An example of a precision sensor used in the voltage standard that requires a coaxial multilayer waveguide can be found in [8]. Another application of coaxial multilayer waveguides are precision high-frequency current shunts [9,10] and AC quantum voltage standards [11]. The latter are used in coaxial structures to deliver microwave power (typically from 20 to 75 GHz) to excite the array of Josephson junction chips. To achieve the high performance of devices (by good impedance matching) or to increase the accuracy of the metrological standard, multilayer constructions require precise knowledge of electrical impedance parameters such as inductance and resistance, which are one of the most important parts of the electrical metrology [12,13,14]. Furthermore, the design and optimization process requires tens of thousands of repetitions when, for example, the Monte Carlo uncertainty evaluation method is used [15]. 

Today, one of the most widely used approaches for determining electrical quantities of cylindrical waveguides is the finite element method (FEM) [16]. This allows computation of the characteristic impedance and its components without using complex equations. However, despite the use of expensive high-performance computing (HPC) systems, waveguide computations take significantly more time than analytical methods. This is the biggest obstacle during optimization and design. Furthermore, even though special meshing techniques provide high precision due to the skin effect, the result is usually not accurate enough [16,17]. The accuracy of waveguide parameter evaluations is particularly important in precision applications, such as calculable thermal converters. These require a knowledge of resistance, inductance and capacitance at the level of ppm (parts per million). This is not possible using approximate formulas [8].

On the other hand, the analytical methods allow much faster evaluations of the waveguide parameters than FEM. Moreover, computation of the characteristic impedance using an analytical algorithm allows for easier implementation of its results in other algorithms, such as the genetic algorithm (GE) [18], to provide Monte Carlo uncertainty evaluations. 

In general, the calculation of the characteristic impedance and its components requires knowledge of the electric and magnetic field distributions inside the inner and outer conductors. These fields are computed from the solutions of the Maxwell differential equations [19]. The solutions are well known in the literature and include so-called Bessel, Hankel, Kelvin or Neumann functions [20]. However, these functions sometimes take particularly high or low values, causing numerical instability. To prevent this instability, research has provided some solutions based on approximations [21,22,23,24,25]. Unfortunately, these algorithms are limited to a specific number of conductive layers, usually one or two. Research into multilayer construction is presented in [26] but does not consider the instability issue. Another approach is the transfer matrix method described in [27]. Numerical stability was improved by replacing Bessel functions with Hankel functions for some algorithms, even though the formula is still unstable at the highest frequencies. A numerically stable solution for a multilayer structure was recently provided in [28]. This algorithm combines the transfer matrix with the scaled modified Bessel function methods. It also allows computation of the internal impedance of the wire and its components at high frequencies. The drawback of this method is that it uses ‘fictive sublayers’ to improve stability in the case of thick conducting layers, thereby increasing the computation time. This disadvantage was circumvented in [29] by creating and solving the linear system of equations without transfer matrices. This provides the fastest and most stable solution of all the given algorithms while maintaining very high precision results. This should be used to compute the internal impedance of the inner multilayer conductor.

However, to the best of the author’s knowledge, there is only one method which allows the internal impedance of the computations of the external (outer) conductor in the coaxial waveguide presented in [30]. Although this method is numerically stable, it requires fictive sublayers for thick conductors, as in [28], thereby increases the computation time. 

The goal of this research is to get rid of the numerical instability for the computation of the multilayer outer conductor parameters of the coaxial cable, based on a linear system of equations. Consequently, the method presented in this article, and in [29], allows the calculation of the characteristic impedance of the cylindrical waveguide for any number of layers with high accuracy and numerical stability, without the necessity to create the fictive layers. The proposed algorithm is validated against the FEM COMSOL Multiphysics software [31] and the method presented in [30]. The presented algorithm can be used in different technical solutions that require rapid and high precision results. 

## 2. Multilayer Waveguide

**Figure 1:**
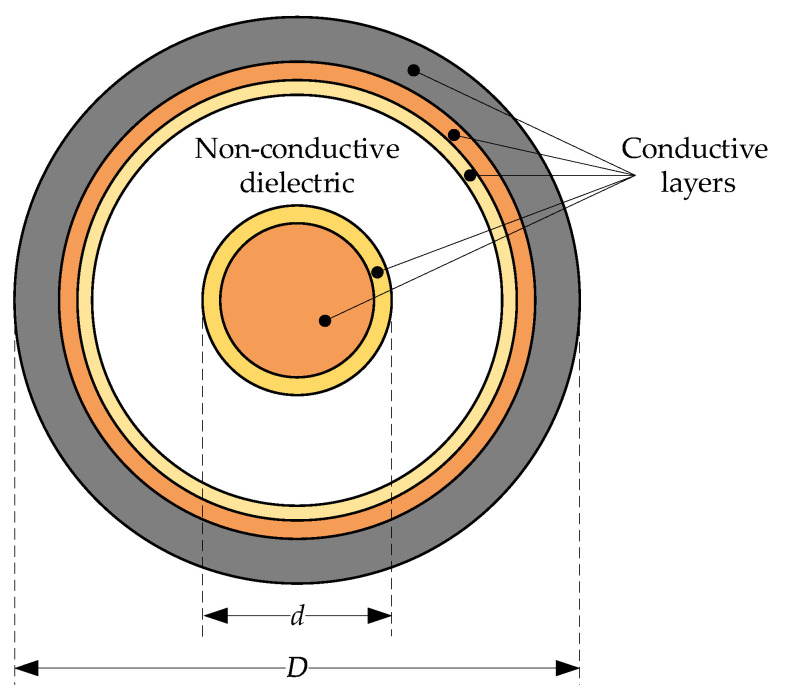
Example of a typical multilayer cylindrical waveguide.

The cross section of a typical multilayer cylindrical structure is presented in Figure 1.

The structure of the assumed coaxial waveguide is composed of two multilayer concentric conductors. The non-identical conductive layers could be responsible for different properties, e.g., stainless steel for mechanical stiffness and gold for oxidation protection. In the case of the waveguide given in Figure 1, the characteristic impedance can be simplified [32]:

(1) $$Z_{c}=\sqrt{\frac{{\bar{Z}}_{i}+{\bar{Z}}_{o}+j\omega L_{s}}{G+j\omega C}},$$

where $L_{s}$ is a self-inductance per unit length, dependent on the outer dielectric diameter D, innermost dielectric diameter d, magnetic permeability constant (in the vacuum) $\mu_{0}$ and relative magnetic permeability $\mu_{r}$ and is described by the equation:

(2) $$L_{s}=\frac{\mu_{0}}{2\pi }\mu_{r}\ln\left(\frac{D}{d}\right).$$

C and G are the capacitance and conductance of the dielectric placed between the inner and outer multilayer conductors, respectively, and are given by:

(3) $$C=\frac{2\pi \varepsilon \prime \varepsilon_{0}}{\ln\left(\frac{D}{d}\right)}$$

and

(4) $$G=\frac{2\pi \omega \varepsilon_{0}\varepsilon \prime \prime }{\ln\left(\frac{D}{d}\right)}$$

where ε′ and ε″ are the real and imaginary part of the electric permittivity, respectively, and $\varepsilon_{0}$ is the permittivity constant (in a vacuum). The symbol ω denotes the angular frequency. 

The internal impedance of the inner conductor ${\bar{Z}}_{i}$ is calculated by the method described in [29]. The equation for calculating the complex internal impedance of the outer multilayer conductor ${\bar{Z}}_{o}$ is developed in Section 3. 

## 3. Internal Impedance of the Outer Conductor

**Figure 2:**
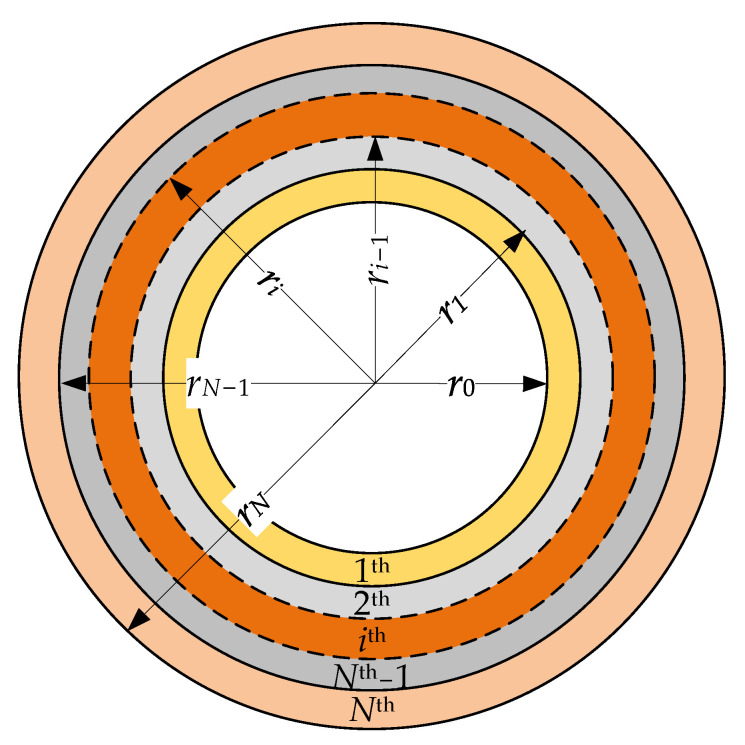
Cross section of the outer conductor of the multilayer coaxial waveguide.

The external multilayer conductor is shown in Figure 2. 

Figure 2 depicts the outer conductor of the multilayer waveguide composed of the *N*^th^ conductive layers, whose *i*^th^ inner and outer boundaries are limited by $r_{i}$ and $r_{i+1}$, respectively. The innermost boundary is indicated by $r_{0},$ while the outermost boundary is $r_{N}$. The physical properties of each *i*^th^ layer are given by electrical conductivity $\sigma_{i}$, absolute magnetic permeability $\mu_{i}$ and absolute electrical permittivity $\varepsilon_{i}$. They allow the computation of the complex wave propagation constant given by [33]:

(5) $${\bar{\gamma }}_{i}=\sqrt{j\sigma_{i}\mu_{i}\omega -\omega^{2}\varepsilon_{i}\mu_{i}}.$$

The magnetic and electric fields for the *i*^th^ layer, at radial distance r from the center of the wire, are expressed by the solutions of the second order Maxwell differential equations in the cylindrical coordinate system [33]:

(6) $${\bar{H}}_{i}=I[{\bar{C}}_{i}\cdot {\bar{I}}_{1}\left({\bar{\gamma }}_{i}\cdot r\right)+{\bar{D}}_{i}\cdot {\bar{K}}_{1}\left({\bar{\gamma }}_{i}\cdot r\right)]$$

(7) $${\bar{E}}_{i}=I\frac{{\bar{\gamma }}_{i}}{\sigma_{i}}\left[{\bar{C}}_{i}\cdot {\bar{I}}_{0}({\bar{\gamma }}_{i}\cdot r)-{\bar{D}}_{i}\cdot {\bar{K}}_{0}({\bar{\gamma }}_{i}\cdot r)\right],$$

where function ${\bar{I}}_{n}$ is the complex-valued modified Bessel function of the first kind of order *n*^th^, ${\bar{K}}_{n}$ is the complex-valued modified Bessel function of the second kind of order *n*^th^, ${\bar{C}}_{i}$ and ${\bar{D}}_{i}$ are complex-valued constants and I is the total current flowing along the longitudinal direction of the waveguide.

As a result of their nature, the implemented modified Bessel functions take very high or low values for high or low electrical conductivity, frequency, magnetic permeability, electric permittivity or radius, respectively. The idea of quasi-linearization of those functions allows them to be rewritten into scaled modified Bessel functions and scaled complex-valued constants, in the form [34]:

(8) $${\bar{I}}_{n}^{s}(x)=exp(-x)\cdot {\bar{I}}_{n}(x)$$

(9) $${\bar{K}}_{n}^{s}(x)=exp(x)\cdot {\bar{K}}_{n}(x)$$

(10) $${\bar{C}}_{i}^{s}=exp({\bar{\gamma }}_{i}r_{i})\cdot {\bar{C}}_{i}$$

(11) $${\bar{D}}_{i}^{s}=exp({\bar{-\gamma }}_{i}r_{i})\cdot {\bar{D}}_{i}$$

Equations (6) and (7), considering Equations (8)–(11), have the form:

(12) $${\bar{H}}_{i}=I\left[{\bar{C}}_{i}^{s}\cdot {\bar{I}}_{1}^{s}({\bar{\gamma }}_{i}\cdot r)+{\bar{D}}_{i}^{s}\cdot {\bar{K}}_{1}^{s}({\bar{\gamma }}_{i}\cdot r)\cdot exp\left({\bar{\gamma }}_{i}\left(r_{i-1}-r\right)\right)\right]$$

(13) $${\bar{E}}_{i}=I\frac{{\bar{\gamma }}_{i}}{\sigma_{i}}\left[{\bar{C}}_{i}\cdot {\bar{I}}_{0}({\bar{\gamma }}_{i}\cdot r)-{\bar{D}}_{i}\cdot {\bar{K}}_{0}({\bar{\gamma }}_{i}\cdot r)\cdot exp\left({\bar{\gamma }}_{i}(r_{i-1}-r)\right)\right].$$

Due to the continuity of the magnetic and electric field in transition between the particular layers, the following expressions for boundary conditions for the *i*^th^ layer are given by:

(14) $${\bar{H}}_{i}\left|\begin{array}{c} r=r_{i} \end{array}={\bar{H}}_{i+1}\left|\begin{array}{c} r=r_{i} \end{array}\right.\right.$$

(15) $${\bar{E}}_{i}\left|\begin{array}{c} r=r_{i} \end{array}={\bar{E}}_{i+1}\left|\begin{array}{c} r=r_{i} \end{array}\right.\right..$$

The outermost and innermost boundary conditions, considering that the current I flows in the opposite direction to the inner conductor of the coaxial waveguide, are denoted by:

(16) $${\bar{H}}_{1}\left|\begin{array}{c} r=r_{0} \end{array}=\frac{-I}{2\Pi r_{0}}\right.$$

(17) $${\bar{H}}_{N}\left|\begin{array}{c} r=r_{N} \end{array}=0\right..$$

By taking the Equations (12) and (13) with internal boundary conditions (14) and (15), the following system of equations can be given for each of the *i*^th^ layers:

(18) $${\bar{C}}_{i}^{s}\cdot {\bar{I}}_{1}^{s}\left({\bar{\gamma }}_{i}\cdot r_{i}\right)+{\bar{D}}_{i}^{s}\cdot {\bar{K}}_{1}^{s}\left({\bar{\gamma }}_{i}\cdot r_{i}\right)\cdot {\bar{\nu }}_{i}-{\bar{C}}_{i+1}^{s}\cdot {\bar{I}}_{1}^{s}\left({\bar{\gamma }}_{i+1}\cdot r_{i}\right)\cdot {\bar{\nu }}_{i+1}+{\bar{D}}_{i+1}^{s}\cdot {\bar{K}}_{1}^{s}\left({\bar{\gamma }}_{i+1}\cdot r_{i}\right)=0$$

(19) $$\frac{{\bar{\gamma }}_{i}}{\sigma_{i}}\left[{\bar{C}}_{i}^{s}\cdot {\bar{I}}_{1}^{s}\left({\bar{\gamma }}_{i}\cdot r_{i}\right)-{\bar{D}}_{i}^{s}\cdot {\bar{K}}_{1}^{s}\left({\bar{\gamma }}_{i}\cdot r_{i}\right)\cdot {\bar{\nu }}_{i}\right]-\frac{{\bar{\gamma }}_{i+1}}{\sigma_{i+1}}\left[{\bar{C}}_{i+1}^{s}\cdot {\bar{I}}_{1}^{s}\left({\bar{\gamma }}_{i+1}\cdot r_{i}\right)\cdot {\bar{\nu }}_{i+1}-{\bar{D}}_{i+1}^{s}\cdot {\bar{K}}_{1}^{s}\left({\bar{\gamma }}_{i+1}\cdot r_{i}\right)\right]=0,$$

where

(20) $${\bar{\nu }}_{i}=exp\left({\bar{\gamma }}_{i}\left(r_{i-1}-r_{i}\right)\right)$$

is the scaling constant. 

Similarly, for the innermost layer, Equation (12) considers the boundary condition (16) in the equation:

(21) $${\bar{C}}_{1}^{s}\cdot {\bar{I}}_{1}^{s}\left({\bar{\gamma }}_{i}\cdot r_{0}\right)\cdot {\bar{\nu }}_{1}+{\bar{D}}_{1}^{s}\cdot {\bar{K}}_{1}^{s}\left({\bar{\gamma }}_{i}\cdot r_{0}\right)=\frac{-I}{2\Pi r_{0}}.$$

Equation (12), for the outermost boundary, considering condition (17), is:

(22) $${\bar{C}}_{N}^{s}\cdot {\bar{I}}_{1}^{s}\left({\bar{\gamma }}_{N}\cdot r_{N}\right)+{\bar{D}}_{N}^{s}\cdot {\bar{K}}_{1}^{s}\left({\bar{\gamma }}_{N}\cdot r_{N}\right)\cdot {\bar{\nu }}_{N}=0.$$

The set of the 2*N* linear Equations (18)–(22) can be computed numerically using MATLAB software or its free equivalent, Octave [35,36]. 

The final complex-valued internal impedance of the outer conductor of the coaxial waveguide is computed as:

(23) $${\bar{Z}}_{o}=\frac{{\bar{E}}_{0}\left|\begin{array}{c} r=r_{0} \end{array}\right.}{I},$$

which, by implementing Equation (7), converts into:

(24) $${\bar{Z}}_{o}=-\frac{{\bar{\gamma }}_{1}}{\sigma_{1}}\left[{\bar{C}}_{1}\cdot {\bar{I}}_{0}\left({\bar{\gamma }}_{1}\cdot r_{0}\right){\bar{\cdot \nu }}_{1}-{\bar{D}}_{i}\cdot {\bar{K}}_{0}\left({\bar{\gamma }}_{i}\cdot r_{0}\right)\right].$$

The resistance R and internal inductance L of this outer tubular conductor can be simply evaluated as:

(25) $$R=Re\{{\bar{Z}}_{o}\}$$

(26) L=Im{Z¯o}ω,

where Re and Im denote the real and imaginary complex impedance ${\bar{Z}}_{o}$, respectively.

## 4. Results

### 4.1. The Validation of the Algorithm

**Figure 3:**
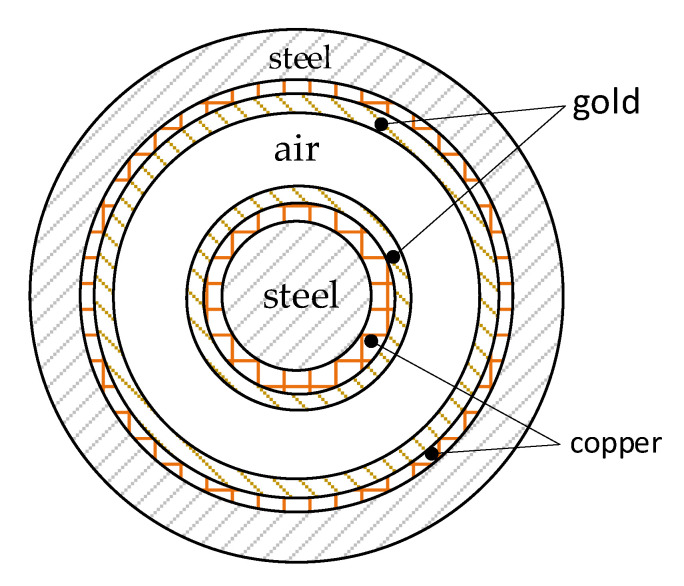
Cross-section of the multilayer waveguide considered in the first validation example.

In the first validation example, the structure of the multilayer coaxial cylindrical waveguide, identical to that in [30], is presented in Figure 3.

This waveguide is assembled from two coaxial cylindrical conductors. The central part of the internal structure is a wire with a radius of 0.7 mm, made of stainless steel with a relative permeability equal to 1.02 and electrical conductivity of 1.32∙10^6^ S/m. The first layer is assumed to be equal to 10 μm thick copper, whose relative permeability is considered to be 0.99994 and electrical conductivity is 5.96 ∙10^7^ S/m. The second material is 5 μm thick gold whose relative permeability is equal to 0.999966 and electrical conductivity is 4.4 ∙10^7^ S/m. The relative permittivity was assumed to be equal to unity for all the materials used. The outer cylindrical conductor is made of the same materials as the inner but in reverse order: the first (counting from the inside) is 5 μm thick gold, then 10 μm thick copper and finally stainless steel pipe, equal to a 100 μm thick wall. The innermost radius of the outer structure is 1.6 mm. The dielectric placed between the conductors is air, whose relative permeability and permittivity is equal to 1.00054 and 1, respectively. The electrical conductivity of air was ignored. 

The equations given in Section 2 and Section 3 were translated into the MATLAB language. The constants and layer thicknesses of the waveguide were used as input data to the MATLAB model. The same data were incorporated into the MATLAB script based on the algorithm presented in [30]. As with those above, the geometry was prepared in the COMSOL Multiphysics FEM software. The results of the module of the characteristic impedance, resistance per unit length (p.u.l.) and inductance p.u.l. are presented in Table 1, Table 2 and Table 3, respectively. 

As presented in Table 1, Table 2 and Table 3, there is no visible difference between the results for |*Z*_c_|, *R* p.u.l. and *L* p.u.l. obtained with the newly presented algorithm and the algorithm from [30]. Both analytical algorithms provide similar results to those obtained from the FEM model for lower frequencies. However, the discrepancy between analytical and FEM results starts to be visible for frequencies higher than 1 MHz, while the skin effect becomes more significant. A detailed discussion of the FEM model at higher frequencies can be found in [37]. The results presented in this subsection confirm the correctness of the algorithm developed. 

### 4.2. Comparison of the Computation Time for the Analytical Algorithms 

The disadvantage of the algorithm presented in [30] is that this two-port network model requires additional fictive sublayers to become stable at higher frequencies, thereby increases the computation time. A comparison of the computation time is now presented. 

**Figure 4:**
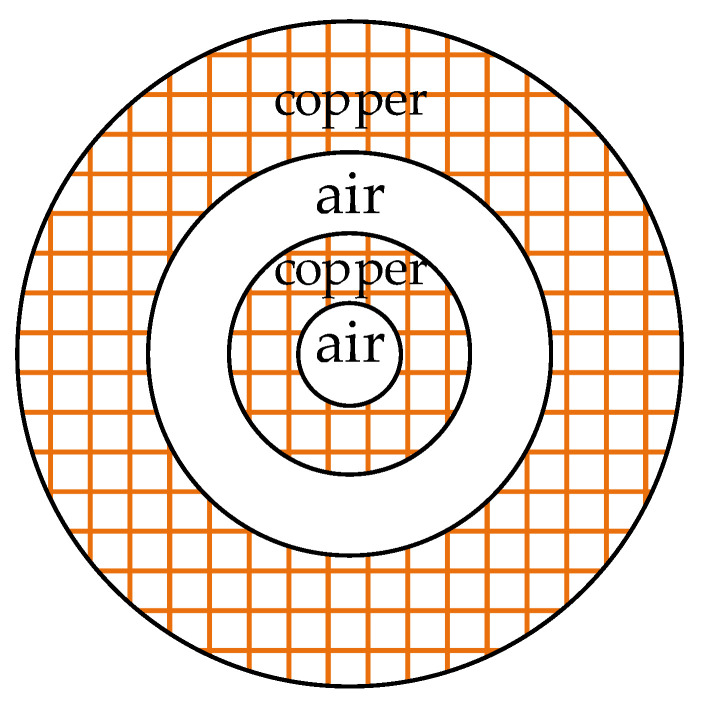
The ‘thick wall’ coaxial waveguide used in example 2.

The waveguide model is depicted in Figure 4. 

**Figure 5:**
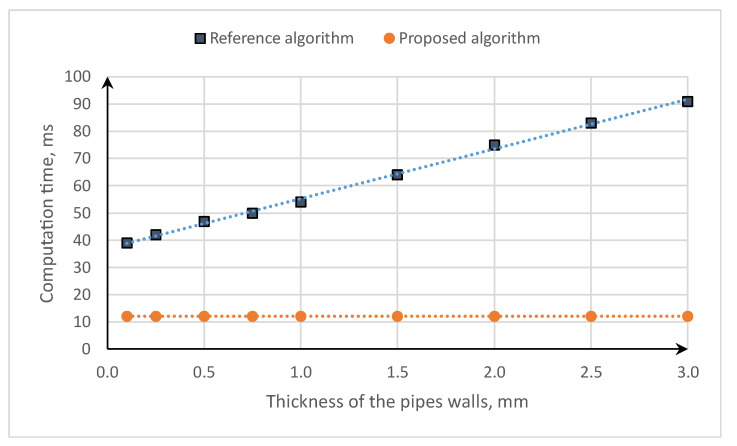
Comparison of the computation time of the characteristic impedance for the algorithm [30] and newly developed equations.

The waveguide used in the second example is assembled from two straight cylindrical concentric copper pipes. The electric and magnetic properties of the copper and air are assumed to be the same as in the first example. The inner radius of the inner pipe is constant and equal to 1 mm. Similarly, the thickness of the dry-air dielectric placed between the conductors is constant and equal to 2 mm. To measure the dependance between the conductor thicknesses and computation time, the wall thickness of both pipes is adjusted in the range from 0.1 to 3.0 mm. The total time is measured for the computation of the characteristic impedance for the frequencies from 1 Hz to 1 GHz, one per decade. The results are displayed in Figure 5. 

Figure 5 shows that the time of the computation of the characteristic impedance increases significantly, from about 39 ms to about 91 ms, for the changes in the conductor thicknesses from 0.1 to 3.0 mm, respectively. This is because it requires an increasing number of fictive sublayers to maintain numerical stability. In contrast, the proposed algorithm takes about 12 ms and does not depend on the conductor thickness. It takes about 69 to 86% less time for the 0.1 and 3.0 mm pipe thickness, respectively. 

## 5. Discussion

The numerical stability of the newly presented formula for the computation of the characteristic impedance of the multilayer waveguide has been improved by two approaches. The first is the scaling of the modified Bessel functions and the scaling of the complex-valued coefficients presented in Equations (8)–(11). These allow for quasi-linearization of the modified Bessel functions, reducing the growing or descending steep slope. The second approach involves the algorithm solving the linear system of equations directly, without performing the transfer matrices, which are necessary in the case of the cascade two port network. Since the number of mathematical operations is lower, the time needed for the computations is also reduced. 

Although both presented analytical algorithms are fast, numerically stable and ultimately precise, the newly developed algorithm would be beneficial for the optimization of complex high frequency devices (e.g., to improve impedance matching) or for uncertainty evaluation by the Monte Carlo method, that usually requires hundreds of thousands of trials. 

## 6. Conclusions

The new robust and numerically stable algorithms presented in this article can be used to calculate the wave impedance and the components of the cylinder coaxial waveguide that form an unlimited number of layers. The analytical formula presented was validated against currently existing algorithms and with FEM COMSOL designs across a wide frequency range, depending on the dimensions of the waveguide. The results of the proposed algorithm are in good agreement with the results obtained by the currently existing analytical model and FEM simulations. 

This paper also shows that, in thick layer calculations, it is not necessary to divide the layers into additional fictional sublayers to ensure numerical stability. The proposed solution allows for computations of the waveguide parameters up to 86% faster than the only currently existing analytical method.

The given solution is especially useful for the optimization of cylindrical complex multilayer structures for wideband calculable thermal converters used in AC-DC voltage transfer, high frequency ultra-precision current shunts and AC quantum voltage standards.

## Figures and Tables

**Table 1:** Comparison of the characteristic impedance for the first validation example.

*f*, Hz	|*Z*_c_|, Ω		
	New Algorithm	Algorithm from [30]	COMSOL FEM
10^0^	25,424	25,424	25,428
10^1^	8039.9	8039.9	8041.1
10^2^	2542.4	2542.4	2543.2
10^3^	804.00	804.00	804.32
10^4^	254.34	254.34	254.31
10^5^	83.227	83.227	83.259
10^6^	50.382	50.382	50.382
10^7^	48.737	48.737	48.742
10^8^	48.534	48.534	48.542
10^9^	48.368	48.368	48.379
4·10^9^	48.318	48.318	48.328

**Table 2:** Comparison of the resistance p.u.l. for the first validation example.

*f*, Hz	*R*, Ω/m		
	New Algorithm	Algorithm from [30]	COMSOL FEM
10^0^	0.2806685	0.2806685	0.2806685
10^1^	0.2806685	0.2806685	0.2806685
10^2^	0.2806685	0.2806685	0.2806685
10^3^	0.2806685	0.2806685	0.2806685
10^4^	0.2806714	0.2806714	0.2806714
10^5^	0.2809538	0.2809538	0.2809538
10^6^	0.3003694	0.3003694	0.3003694
10^7^	0.3733909	0.3733909	0.3733909
10^8^	0.8968540	0.8968540	0.8968594
10^9^	3.0633201	3.0633201	3.0635652
4·10^9^	9.6498060	9.6498060	9.6366165

**Table 3:** Comparison of the inductance p.u.l. for the first validation example.

*f*, Hz	*L*, nH/m		
	New Algorithm	Algorithm from [30]	COMSOL FEM
10^0^	170.8578	170.8578	170.8578
10^1^	170.8578	170.8578	170.8578
10^2^	170.8578	170.8578	170.8578
10^3^	170.8578	170.8578	170.8578
10^4^	170.8575	170.8575	170.8575
10^5^	170.8273	170.8273	170.8273
10^6^	168.7718	168.7718	168.7718
10^7^	164.0388	164.0388	164.0388
10^8^	162.7767	162.7767	162.7769
10^9^	161.6674	161.6674	161.6794
4·10^9^	161.3358	161.3358	161.3464

## Data Availability

No associated data.
